# ADAPTING A NOVEL BEHAVIORAL TEST TO SCREEN FOR PRECLINICAL ALZHEIMER’S DISEASE IN PRIMARY CARE

**DOI:** 10.1093/geroni/igae098.2845

**Published:** 2024-12-31

**Authors:** Sydney Schaefer, Elizabeth Fauth, Andrew Hooyman, Jill Love

**Affiliations:** Arizona State University, Tempe, Arizona, United States; Utah State University, Logan, Utah, United States; Arizona State University, Tempe, Arizona, United States; Neurosessments LLC, Tempe, Arizona, United States

## Abstract

Most early dementia cases are missed in primary care, due partly to subjective screening (i.e., self-reported concerns, clinician observation). Objective tests (e.g., Mini-Mental Status Exam) are valid but take too long for widespread use in primary care. Extensive research on our qBEANS behavioral test supports that it is sensitive and specific to Alzheimer’s disease pathology, and is prognostic of cognitive and functional decline. This timed test engages motor learning, visuospatial memory, and executive function, involving six trials of 15 repetitions in which patients spoon raw kidney beans to small plastic cups in a sequence; no technology or wearable sensors are required. However, the current version is too long for primary care adoption (~7 minutes). The purpose of this study was to identify the minimum number of trials needed for reliability relative to the original longer version. Forty-eight non-demented participants (mean±SD age: 75.4±7.0 years; 77% female) completed the original version while the duration of each repetition was also recorded. Our reliability threshold was an intraclass correlation (ICC) >0.75. Results indicated that the most reliable shorter version involved only three full (15-repetition) trials (mean completion time = 232 seconds, or 3.85 minutes), 48% faster than the original version (ICC=0.85). While this shorter version is reliable, future work will determine if it is acceptable in primary care settings when administered by medical assistants during the rooming process. Findings also warrant further development of qBEANS as a direct-to-consumer product, given its low cost and ease of administration.

